# Placental peptides metabolism and maternal factors as predictors of risk of gestational diabetes in pregnant women. A case-control study

**DOI:** 10.1371/journal.pone.0181613

**Published:** 2017-07-21

**Authors:** Robert Amadu Ngala, Linda Ahenkorah Fondjo, Peter Gmagna, Frank Naku Ghartey, Martin Akilla Awe

**Affiliations:** 1 Department of Molecular Medicine, School of Medical Science, Kwame Nkrumah University of Science and Technology (KNUST), Kumasi, Ghana; 2 Department of Chemical Pathology, University of Cape Coast, Cape Coast- Ghana; University of Missouri Columbia, UNITED STATES

## Abstract

**Background:**

Gestational diabetes is a risk factor for perinatal complications; include shoulder dystocia, birth injuries such as bone fractures and nerve palsies. It is associated with later development of type 2 diabetes, the risk of macrosomia and other long-term health effects of infants born to diabetic mothers. The study assesses placental peptides and maternal factors as potential predictors of gestational diabetes among pregnant women.

**Material and methods:**

A total of 200 pregnant women were recruited for the study, 150 pregnant women without pre gestational diabetes including 50 women with low risk factors of diabetes as controls and 50 other pregnant women with pregestational diabetes as control. Fasting blood glucose and the lipid profile were determined by enzymatic methods using Envoy^®^ 500 reagents (Vital Diagnostics, USA). Glycated haemoglobin was assessed using the Cation Exchange resin method. Leptin and the Human Placenta Lactogen were assayed using the Sandwich-ELISA technique. Beta chorionic gonadotrophin, insulin, progesterone and estradiol were determined using chemilumiscence imunoassay technique on MAGLUMI 600 analyzer. Anthropometry, including BMI and blood pressure were also measured.

**Results:**

Fasting plasma glucose (FBG), insulin, insulin resistance, glycated haemoglobin and Human Placenta Lactogen(HPL)were significantly (p**<**0.0001) increased in the pregestational diabetic women whereas progesterone and estradiol were significantly decreased. In the second trimester however, there was no significant difference (p>0.05) in estradiol, insulin, insulin resistance and HPL between the pregnant women who developed gestational diabetes and those who did not. Leptin, progesterone and FBG were significantly increased in those who developed GDM. The risk of developing gestational diabetes increased with overweight (OR = 1.76, P = 0.370) and family history of diabetes (OR = 2.18, P = 0.282).

**Conclusion:**

Leptin, progesterone, estradiol estimated in this study were increased in the gestational diabetes mellitus women and fairly predicted gestational diabetes in the non-diabetics pregnant women. Obesity, aging and family history of diabetes were strongly predictive of gestational diabetes.

## Introduction

In a normal pregnancy, sensitivity to insulin reduces with increasing gestation age [1].It is a physiological programming designed to shift metabolic fuel to the fetus for development [2]. In this state, insulin sensitivity in pregnant non-diabetics is reportedly reduced [3].The decrease in insulin sensitivity with progression in pregnancy is due partly to the inhibitory effect of placenta peptides (C-reactive protein, leptin and human placental lactogen etc) on insulin secretion [4,5].The effects of these placenta peptides and hormones cause the enlargement of the islets of Langerhans cells and or the hyperplasia of the pancreatic β-cells[6] to increase the secretion of more insulin, resulting in compensated hyperinsulinaemia [7]. Obesity and chronic insulin resistance during pregnancy are most common factors that exacerbate β-cell dysfunction [8]. Gestational diabetes mellitus (GDM) occurs when the pancreatic β-cells are unable to produce sufficient insulin to offset the persisting insulin resistance in pregnant women [3].

Similar frequencies of HLA-DR2, DR3 and DR4 antigens in GDM as in healthy pregnant women have been reported and this has been found to be associated with low prevalence of autoimmune destruction of β-cells and therefore rules out the possibility that GDM is a disease of autoimmune origin [9].

Gestational diabetes mellitus is reported in 2 to 9 percent of all pregnancies,[10] it is a risk factor for adverse perinatal complications that may later result in the development of type 2 diabetes.[11]. The long-term health effects on infants born to mothers with gestational diabetes include impaired glucose tolerance,[12] obesity[13] and impaired intellectual ability [14].

Pregnant women with high risk factors for GDM or showing clinical signs of diabetes are usually subjected to an oral glucose tolerance test (OGTT) at first trimester and then again between 24 and 28 weeks if the initial tests were negative. At weeks 24–28 of gestation, it is recommended that all women undergo an OGTT. GDM is defined as a fasting plasma glucose level ≥5.5 mmol/l and/or a 2-h plasma glucose level ≥8.5 mmol/l after a fasting 75-g OGTT [15]. Treatment of gestational diabetes reduces perinatal morbidity and may also improve the woman’s health-related quality of life [16].

Screening for GDM is limited, the standard screening procedure for diabetes and pre-diabetes, such as fasting blood glucose and glycated haemoglobin, are not recommended for screening for GDM. At first trimester FBG alone does not distinguish between GDM, impaired glucose tolerance or preexisting diabetes, also HbA1c predicts glucose metabolic state over a period of 3 months and therefore does not distinguish between preexisting diabetes and GDM. However, the recommended method is an oral glucose tolerance test (OGTT) [17] and this is expensive, invasive, and requires hospital visit and multiple blood draws.

The development of abnormal glucose metabolism after gestational diabetes can be predicted readily by means of available clinical and anthropometric measurements.[18, 19] Similarly, if there were a proper assessment of the various risks as well as markers that may predict the onset of gestational diabetes among pregnant women, it would in turn allow practitioners to better manage patients and prevent many pregnancy associated complications. The current study therefore was aimed at assessing the roles of placental peptides and maternal factors as potential predictors of gestational diabetes among pregnant women in the Tema Metropolis of Ghana.

## Methods

A cross-sectional case-control study was carried out at the Tema General Hospital, Tema Polyclinic and Provita Specialist Hospital all in Ghana, from March 2014 to March 2015. A total of 200 pregnant women aged between 17–45 years and mean age of 28.43±4.95 years were recruited for the study, comprising of 150 pregnant women without pregestational diabetes (subjects) including 50 women in the group with low risk factors of diabetes as controls. Fifty (50) other pregnant women with pregestational diabetes were recruited as controls.

### Study participant selection

To determine gestational diabetes in pregnant women, a standard OGTT was performed between the 24^th^ and 28^th^ week of gestation after an overnight fast (8–12 hours), by administering 75 g anhydrous glucose in 300 ml water. Women were advised to follow a normal diet 48 hours before the oral glucose-tolerance test (OGTT). However, pregnant women belonging to high risk diabetes population were screened during the first trimester in order to detect a previously undiagnosed diabetes mellitus. Gestational age was also determined on the basis of the woman’s last normal menstrual period and if it coincided within 1week of the date determined by ultrasound done between 16^th^and 20^th^weeks of gestation, otherwise the ultrasound estimates was used. A pretested guided questionnaire was administered to collect demographic data of family history of diabetes, parity, miscarriages, and complications of pregnancy. Anthropometric parameters including BMI and blood pressures were measured. Pregnant women who reported at the antenatal clinic were included; unless they opted out of the study, non pregnant women seeking fertility advice were excluded from the study.

### Ethical clearance approval

Ethical clearance was obtained from the Committee on Human Research and Publications Ethics (CHRPE)(REF) of the School of Medical Sciences (SMS), Kwame Nkrumah University of Science & Technology (KNUST).

### Informed consent of subjects

Clients who reported at the antenatal clinic and agreed to participate in the study gave written informed consent after detailed explanation of the study procedures had been given to them before the recruitment into the study. Guardians of participants below 18 years of age gave consent on their behalf.

### Sample collection

An over-night (8–12hr) fasted sample of 6ml venous blood sample was taken from subjectsduring the first trimester and between the 24^th^ and 28^th^ week of gestation. The sample was dispensed into gel separator tubes (yellow top), EDTA (mauve top) and Fluoride tubes (for glucose) for analysis. Blood samples were allowed to clot. After clotting, samples were centrifuged at 3000 g for 4 min and the sera separated into appropriate tubes and frozen at -20°C until analysis. Metabolites analyzed for included total cholesterol, triglyceride (TG), low-density lipoprotein (LDL), high-density lipoprotein (HDL), fasting blood glucose serum insulin, glycosylated haemoglobin, leptin, estradiol, progestrone, Human placenta lactogen and beta HCG. The homeostasis model assessment index-insulin resistance (HOMA-IR), based on fasting insulin and glucose measured in a single blood sample, was used to calculate insulin resistance [20].

### Biochemical analysis

The frozen serum samples were thawed to room temperature before being analyzed.

### Lipid profile

Triglycerides, total cholesterol and HDL-cholesterol were assayed using enzymatic methods with Envoy^®^ 500 reagents (Vital Diagnostics, USA) according to the manufacture’s specification on BT 5000^®^ Random Access Chemistry Analyzer (Biotecnica, Italy).

### Glycated haemoglobin

The Cation Exchange Resin method for the selective estimation of glycated haemoglobin (HbA1c) method in human whole blood was used, on Microtech 3000 semi auto analyzer.

### Leptin

Maternal serum leptin concentration was measured quantitatively by Sandwich-ELISA technique using Leptin (Human LEP) kit (Elabscience Biotechnology co ltd WuHan P.R.C). The lowest limit of detection of the assay was 0.094ng/mL. The intra assay and inter assay CV ranged from 2.5% to 9.5% and from 4.7% to 8.3%, respectively.

### Human placenta lactogen (HPL)

Maternal serum Human placenta lactogen concentration was measured quantitatively by Sandwich-ELISA technique on Human Reader HS, a semi auto analyzer.

### Progestrone (P4) and estradiol (E2)

Progestrone and Estradiol, were determined using chemilumiscence imunoassay technique on MAGLUMI 600 analyzer. The method uses ABEI label anti- E2/anti-prog antibody and a purified E2/ Prog antigen to coat magnetic microbeads. The ABEI labeled microbeads were incubated at 37^°^C forming antigen antibody complex which was precipitated in a magnetic field and the supernatant descanted. The addition of starter I and 2 initiates chemilumiscence reaction, and the light signal produced measured.

### Beta chorionic gonadotrophin (β-HCG) and Insulin assay

Beta chorionic gonadotrophin and insulin were also determined using MAGLUMI 600 analyzer. The sandwich chemilumiscence imunoassay technique was used, adhering to the manufacturers’ protocol.

### Statistical analysis

Results were expressed as mean ± S.D and statistical analysis performed using SPSS version 20.0 (SPSS Inc.) and GraphPad prism 5 for Windows. Normal distribution and homogeneity of the variances were tested using Kolmogorov-Smirnov and Levène tests, respectively. Student t-test was used to compare the significance of the difference in the mean values of any two groups and chi square analysis was used to compare frequency between the two groups. Linear regression analysis was used to study the correlation between the parameters. Correlations between parameters were analyzed using the Pearson R test for variables with normal distribution and ROC to determine the sensitivity and specificity of FBG, HbA1c, Insulin and β-HCG as markers of GDM. P<0.05 was considered statistically significant.

## Results

Table 1 show 50 women with pre-gestational diabetes (controls) and 150 pregnant women without diabetes (cases) during first trimester, 50 of which have very low risk of developing diabetes. Fasting plasma glucose, insulin, insulin resistance, glycated haemoglobin, and Human Placenta Lactogen were significantly increased (p**<**0.0001) in the pregestational diabetic controls, whereas, progesterone and estradiol were significantly increased in the nondiabetic pregnant women. BMI was also significant (p**<**0.05) increased in pregnant women with pregestational diabetes(Table 1).

**Table 1:** Biochemical characteristics of non-diabetic pregnant women (Cases) and pregnant women with regestational diabetes (control) in the first trimester.

Variable	Cases	Controls	P-value
	(n = 150)	(n = 50)	
**Age (Mean**± SD)	29.08±5.26	27.78±4.67	0.136
**FBG (mmol/l)**	4.28 ± 0.91	5.86 ± 2.03	**<0.001**
**HB (mg/dl)**	10.28 ± 1.83	10.32 ± 1.72	0.886
**HbA1c (%)**	4.70 ± 1.53	5.56 ± 1.12	**<0.0001**
***Blood Pressure (mmHg)***			
SBP	114.91 ± 6.07	115.34 ± 6.59	0.669
DBP	82.66 ± 7.82	82.88 ± 7.78	0.861
**BMI n (Kg/m**^**2**^**)**	23.29 ± 3.40	24.75 ± 3.51	**0.010**
**BMI n (%)**			0.161
Underweight	2 (1.3)	0 (0.0)	
Normal	97 (64.7)	28 (56.0)	
Overweight	48 (32.0)	18 (36.0)	
Obese	3 (2.0)	4 (8.0)	
**TC (mmol/L)**	4.97 ± 1.36	5.25 ± 1.02	0.194
**TG (mmol/L)**	1.32 ± 0.79	1.45 ± 0.89	0.354
**β-HCG**	4880.07 ± 554.13	4923.04 ± 390.67	0.612
**Insulin (μIU/mL)**	14.43 ± 7.76	18.51 ± 9.30	**0.003**
**IR (μIU/mL)**	2.75 ± 1.76	4.81 ± 2.90	**<0.0001**
Leptin (ng mL^-1^)	24.12 ± 9.6	22.89 ± 9.4	0.232
**P4** (ng/ml)	60.50 ± 21.91	44.86 ± 14.82	**<0.0001**
**E2** (pmol/L)	4249.94 ± 1592.88	2470.52 ± 1297.95	**<0.0001**
**HPL** (μg/ ml)	75.70 ± 23.46	105.11 ± 21.35	**<0.0001**

p < 0 .05 was considered statistically significant

FBG;fasting blood glucose, HB; haemoglobin, HbA1c; glycated haemoglobin, SBP; systolic blood pressure, DBP; diastolic blood pressure, BMI; body mass index, TC; total cholesterol, TG; triglyceride, β-HCG; beta-human chorionic gonadotropin, IR; insulin resistant, P4; Progesterone, E2; Estradiol, HPL; Human Placenta Lactogen

After screening for gestational diabetes among the pregnant women without diabetes (n = 150), between 24–28 week gestation,12 of them developed GDM giving a prevalence of 8% GDM. Fasting plasma glucose, glycated haemoglobin, leptin, and progesterone were significantly (p<0.0001,p = 0.029, p<0.0001,p = 0.043 respectively) increased in the pregnant women who developed GDM. Estradiol and HPL were non significantly changed in the GDM women, whilst plasma haemoglobin was higher in the women without GDM (Table 2).

**Table 2:** Comparison of biochemical variables in case subjects who developed GDM and those without GDM in the second trimester.

Variable	GDM	No GDM	P-value
	(n = 12)	(n = 138)	
**FBG (mmol/l)**	6.33 ± 1.03	4.27 ± 0.90	**<0.0001**
**HB (mg/dl)**	9.23 ± 2.83	10.37 ± 1.70	**0.035**
**HbA1c (%)**	5.62 ± 2.92	4.62 ± 1.33	**0.029**
**Glycemic control**			**0.007**
≤ 7% (Good)	10 (83.3)	135 (97.8)	
> 7% (Poor)	2 (16.7)	3 (2.2)	
**Blood Pressure (mmHg)**			
SBP	115.00 ± 4.99	114.90 ± 6.17	0.955
DBP	82.42 ± 7.45	82.68 ± 7.88	0.913
**BMI n (Kg/m**^**2**^**)**	21.8 ± 2.85	23.41 ± 3.42	0.116
**BMI n (%)**			0.118
Underweight	1 (8.3)	1 (0.7)	
Normal	6 (50.0)	91 (65.9)	
Overweight	5 (41.7)	43 (31.2)	
Obese	0 (0.0)	3 (2.2)	
**TC (mmol/L)**	5.09 ± 0.81	4.96 ± 1.40	0.751
**TG (mmol/L)**	1.53 ± 0.11	1.31 ± 0.77	0.363
**β-HCG**	5000.0 ± 0.00	4869.65 ± 576.70	0.436
**Insulin (0μIU/mL)**	17.17 ± 11.38	14.30 ± 7.37	0.221
**IR (μIU/mL)**	3.50 ± 2.75	2.68 ± 1.64	0.120
**leptin** (ng mL^-1^)	36.5 ± 9.2	19.8 ± 9.0	**<0.0001**
**P4** (ng/ml)	72.76 ± 11.54	59.43 ± 22.30	**0.043**
**E2** (pmol/L)	4502.01 ± 1338.39	4228.02 ± 1615.44	0.569
**HPL** (μg/ ml)	66.45 ± 27.43	76.50 ± 23.02	0.155

p < 0 .05 was considered statistically significant

FBG; fasting blood glucose, HB; haemoglobin, HbA1c; glycated haemoglobin, SBP; systolic blood pressure, DBP; diastolic blood pressure, BMI; body mass index, TC; total cholesterol, TG; triglyceride, β-HCG; beta-human chorionic gonadotropin, P4; Progesterone, E2; Estradiol, HPL; Human Placenta Lactogen

**Fig 1:**
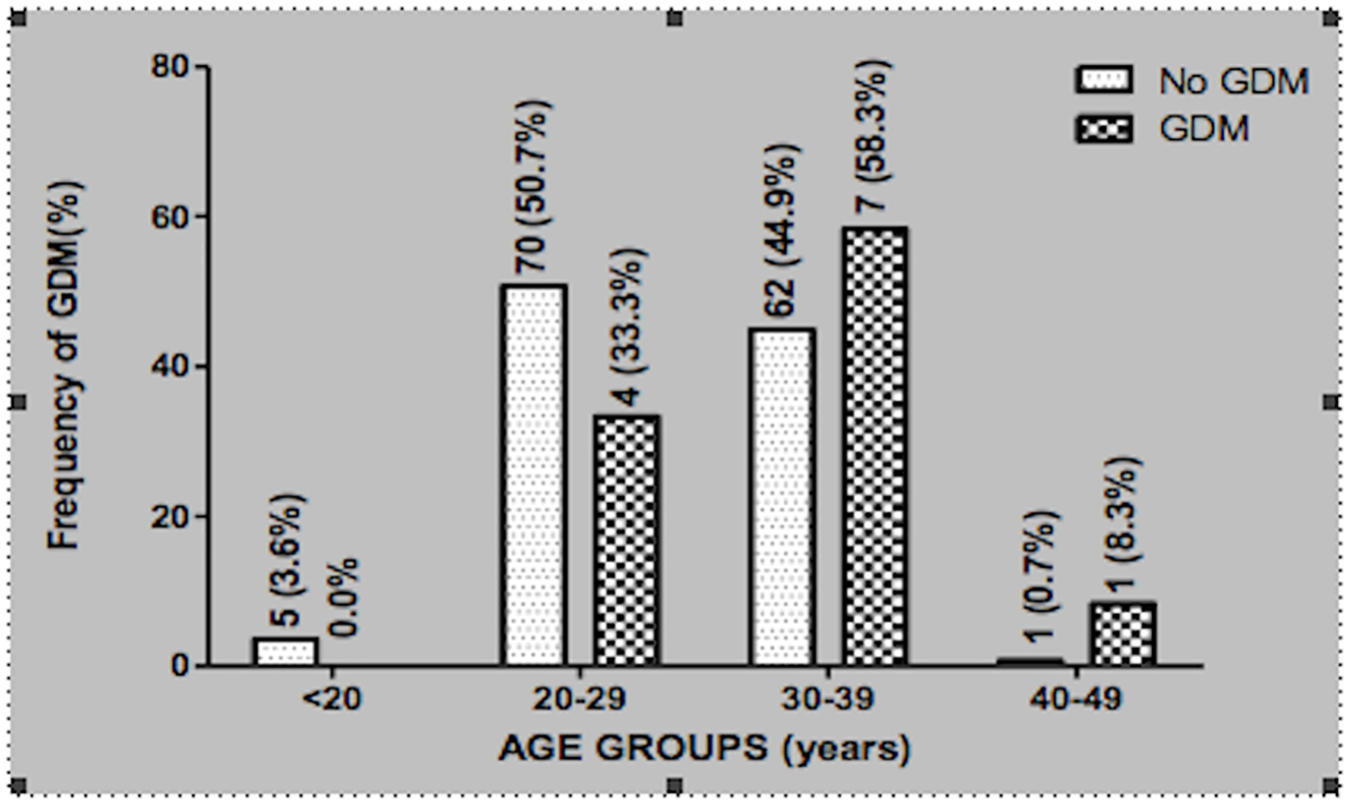
Prevalence of GDM in the various age groups of the pregnant women.

When the pregnant women were stratified according to age, pregnant women less than 20yrs of age did not present with GDM, whilst, 33.3% of them in the age range of 20–29yrs had GDM. The highest prevalence of 58.3% GDM occurred at 30–39yrs, whilst only 8.3% GDM was found in the 40–49yrs range (Fig 1)

Pregnant women who developed GDM had significantly (p<0.01) raisedfasting blood glucose, (p<0.014), BMI (P = 0.022),leptin, progesterone, estradiol (p<0.0001) than the pregnant women with low risk of diabetes (Table 3)but significantly low(p<0.0001) human placenta lactogen levels.

**Table 3:** Biochemical comparison between pregnant women who developed GDM (cases) and pregnant women withlow risk of diabetes (controls).

Variable	GDM	Controls	P-value
	(n = 12)	(n = 50)	
**FBG (mmol/l)**	5.86 ± 2.03	4.33± 1.03	**0.014**
**HB (mg/dl)**	9.22 ± 2.83	10.32 ± 1.72	0.086
**HbA1c (%)**	5.62 ± 2.92	5.56 ± 1.11	0.900
**Glycemic control**			0.223
≤ 7%	10 (83.3)	47 (94.0)	
> 7%	2 (16.7)	3 (6.0)	
**Blood Pressure (mmHg)**			
SBP	115.00 ± 4.99	115.34 ± 6.59	0.868
DBP	82.42 ± 7.45	82.88 ± 7.78	0.853
**BMI n (Kg/m**^**2**^**)**	21.80 ± 2.85	20.12 ± 2.40	**0.022**
**BMI n (%)**			0.153
Underweight	1 (8.3)	5 (10.0)	
Normal	4 (33,3)	30 (60.0)	
Overweight	5 (41.7)	15 (30.0)	
Obese	2 (16.6)	0 (0.0)	
**TC (mmol/L)**	5.09 ± 0.812	5.25 ± 1.23	0.630
**TG (mmol/L)**	1.53 ± 1.11	1.45 ± 0.89	0.799
**β-HCG**	5000.00 ± 0.00	4923 ± 390.67	0.500
**Insulin (μIU/mL)**	17.17 ± 11.38	18.51 ± 9.30	0.668
**IR (μIU/mL)**	3.50 ± 2.75	4.81 ± 2.90	0.163
**leptin** (ng mL^-1^)	37.5 ± 7.2	18.8 ± 6.0	<**0.0001**
**P4** (ng/ml)	72.76 ± 11.54	44.86 ± 14.82	**<0.0001**
**E2**(pmol/L)	4502.01 ± 1338.39	2470.52 ± 1297.95	**<0.0001**
**HPL** (μg/ ml)	66.45 ± 27.43	105.11 ± 21.35	**<0.0001**

Comparison between means was done using un-paired t-test. p < 0 .05 was considered statistically significant

**FBG;fasting blood glucose, HB;** haemoglobin, **HbA1c; glycated** haemoglobin **SBP; systolic blood pressure, DBP; diastolic blood pressure, BMI; body mass index, TC; total cholesterol, TG; triglyceride, β-,IR:insulin resistant. β-HCG; beta-human chorionic gonadotropin, P4; Progesterone, E2; Estradiol, HPL; Human Placenta Lactogen**

Table 4 shows the Accuracy of diagnosis of first trimester FBG-1, FBG-2, HbA1c, Insulin, β-HCG, P4, E2 and HPL.

**Table 4:** Accuracy of first trimester glycated haemoglobin and placental peptides as markers of gestational diabetes.

Threshold values	Sensitivity (95% CI)	Specificity (95% CI)	AUC (95% CI)	P-value
**GDM**				
**FBG 1(mmol/L) 3.75**	33.3% (9.93–65.1)	72.0% (64.2–79.7)	0.49 (0.31–0.67)	0.928
**FBG 2 (mmol/L) 5.6**	100% (73.5–100)	95.7% (90.8–98.4)	0.99 (0.97–1.00)	**<0.0001**
**HBA1c (%) 5.6**	33.3% (9.93–65.1)	87.0% (80.2–92.1)	0.53 (0.33–0.72)	0.8004
**Insulin (μIU/mL) 28.5**	41.7% (15.2–72.3)	94.1% (88.7–97.4)	0.51 (0.28–70)	0.9244
**B-HCG 4947**	100% (73.5–100)	7.65 (3.0–12.0)	0.53 (0.37–0.70)	0.7084
**P4 74.95**	58.3% (27.7–84.8)	71.0%(62.7–78.4)	0.67 (0.54–0.79)	**0.050**
**E2 5256**	50.0% (21.1–78.9)	63.0% (54.4–71.1)	0.53 (0.38–0.68)	0.737
**HPL 65.61**	50% (21.1–78.9)	71.7 (63.5–79.1)	0.59 (0.39–0.79)	0.305

**FBG;fasting blood glucose, HB;** haemoglobin, **HbA1c; glycated** haemoglobin, **SBP; systolic blood pressure, DBP; diastolic blood pressure, BMI; body mass index, TC; total cholesterol, TG; triglyceride, β-HCG; beta-human chorionic gonadotropin,IR:insulin resistant, P4; Progesterone, E2; Estradiol, HPL; Human Placenta Lactogen**

The diagnostic value of 3.75 mmol/L for first trimester fasting blood glucose (FBG-1) had a sensitivity of 33.3% and Specificity of 72.0% as marker for GDMand5.6 mmol/L diagnostic value with significant (p**<**0.0001) 100% sensitivity and 95.7% specificity for second trimester fasting blood glucose (FBG-2). First trimester HbA1c with sensitivity of 33.3% and specificity of 87.0% had a threshold value of 5.6%. β-HCG showed 100% sensitivity and specificity of 7.65% at 4947. E2 and HPL showed similar sensitivity of 50.0% but difference in specificity of 63.0% and 71.7% at 5256 and 65.61 respectively.

The risk of developing GDM increased with age (OR = 1.02, P = 0.757). Overweight (OR = 1.76, P = 0.370) and family history of diabetes (OR = 2.18, P = 0.282) (Table 5). Pregnant women in the first trimester, with a history of abortion, miscarriage and pre-mature birth as complications had higher risk of developing GDM. Also the risk of developing GDM increased with increasing plasma progesterone.

**Table 5:** Logistic regression for predictors of gestational diabetes mellitus.

Predictors	OR (95% CI)	P-value
**Age (years)**	1.02 (0.91–1.13)	0.757
**SBP (mmHg)**	1.00 (0.91–1.10)	0.963
**DBP (mmHg)**	0.99 (0.92–1.07)	0.897
**BMI n (%)**		
Underweight	15.17 (0.84–27.35)	0.065
Normal*	Reference	
Overweight	1.76 (0.51–6.10)	0.370
Obese		
**Family History of other conditions**		
None*	Reference	
Diabetes	2.18 (0.53–9.06)	0.282
Obesity	-	-
Hypertension	-	-
Thyroid disease	0.77 (0.09–6.01)	0.814
**History of Full term delivery**		
Yes	0.59 (0.18–1.97)	0.393
No*	Reference	
**History of Abortions**		
Yes	1.86 (0.45–7.43)	0.382
No*	Reference	
**History of Miscarriages**		
Yes	1.30 (0.15–11.25)	0.810
No*	Reference	
**History of Pregnancy Complications**		
None	Reference	
Pre-eclampsia	-	-
Pre-mature birth	1.27 (0.15–10.99)	0.826
Still birth	-	-
β-HCG	1.12 (0.00–4.85)	0.994
Insulin (μIU/mL)	1.04 (0.97–1.12)	0.256
**P4** (ng/ml)	1.04 (1.00–1.09)	**0.050**
**E2** (pmol/L)	1.00 (1.00–1.00)	0.707
**HPL** (μg/ ml)	0.98 (0.95–1.01)	0.105

SBP; systolic blood pressure, DBP; diastolic blood pressure, BMI; body mass index, β-HCG; beta-human chorionic gonadotropin P4; Progesterone, E2; Estradiol, HPL; Human Placenta Lactogen

Body mass index was significantly and positive correlated with Glycated haemoglobin(.702*). Insulin resistance was positively associated with insulin concentration (.922**) and FBG (.714**) in the women with GDM. Glycated haemoglobinwas positively associated with fasting blood glucose (-.510**) but negatively associated with β-HCG (-303*) (Table 6)and fasting blood glucose levels negatively associated with β-HCG (-.469**) and progesterone (-298*)

**Table 6:** Relationship between glycated haemoglobin, placenta peptides and dyslipidemia among pregnant women with GDM (upper portion) and those without diabetes (lower portion).

Variables		HbA1c	FBG	β-HCG	Insulin	IR	BMI	P4	HPL	E2	TC	TG
**HbA1c**	r	1	0.25	.^a^	-0.096	-0.087	.702*	0.073	-0.167	0.352	-0.104	0.555
	P-value		0.434	.	-0.766	0.788	0.011	0.821	0.604	0.261	0.748	0.061
**FBG**	r	.510**	1	.^a^	0.427	.714**	0.163	-0.409	-0.518	-0.13	-0.369	0.244
	P-value	0		.	0.166	0.009	0.613	0.187	0.084	0.686	0.237	0.445
**β-HCG**	r	-.303*	-.469**	.^a^	.^a^	.^a^	.^a^	.^a^	.^a^	.^a^	.^a^	.^a^
	P-value	0.033	0.001		.	.	.	.	.	.	.	.
**Insulin**	r	-0.125	-0.014	0.071	1	.922**	-0.097	-0.293	-0.314	-0.448	-0.144	-0.238
	P-value	0.388	0.921	0.622		0	0.763	0.356	0.32	0.144	0.655	0.457
**IR**	r	0.193	.545**	-0.172	.803**	1	-0.084	-0.357	-0.403	-0.455	-0.223	-0.159
	P-value	0.178	0	0.231	0		0.796	0.254	0.194	0.138	0.486	0.623
**BMI**	r	-0.008	-0.105	-0.237	-0.17	-0.176	1	0.142	-0.161	0.576	0.024	0.438
	P-value	0.958	0.47	0.097	0.239	0.221		0.659	0.618	0.05	0.942	0.154
**P4**	r	-0.144	-.298*	0.162	0.237	0.02	-0.092	1	0.524	0.235	0.003	-0.212
	P-value	0.318	0.035	0.261	0.098	0.891	0.525		0.08	0.461	0.994	0.509
**E2**	r	0.142	0.066	0.133	-0.206	-0.155	0.155	0.014	-0.282	1	0.076	0.372
	P-value	0.325	0.651	0.357	0.152	0.284	0.282	0.924	0.374		0.814	0.234
**HPL**	r	0.114	-0.063	0.077	-0.114	-0.117	0.058	0.149	1	-0.219	-0.247	-0.551
	P-value	0.431	0.666	0.593	0.431	0.42	0.69	0.3		0.126	0.439	0.064
**TC**	r	0.18	0.12	-0.144	0.129	0.144	0.172	0.027	0.126	0.138	1	0.413
	P-value	0.211	0.407	0.318	0.372	0.318	0.232	0.851	0.384	0.341		0.182
**TG**	r	0.186	0.011	-0.027	-0.011	-0.004	0.052	-0.203	0.041	.314*	0.187	1
	P-value	0.196	0.938	0.852	0.941	0.98	0.717	0.158	0.777	0.026	0.193	

*R* = Correlation coefficient

* Correlation is significant at the 0.05 level (2-tailed)

** Correlation is significant at the0.01 level (2-tailed)

^a^not be computed because at least one of the variables is constant.

**FBFBG;fasting blood glucose, HB;** haemoglobin, **HbA1c; glycated** haemoglobin, **SBP; systolic blood pressure, DBP; diastolic bloblood pressure,BMI; body mass index, TC; total cholesterol, TG; triglyceride, β-HCG;beta-human chorionic gonadotropin; P4; Progesterone, E2; Estradiol, HPL; Human Placenta Lactogen**

## Discussion

Pregnancy has been identified as a diabetogenic condition characterized by the development of insulin resistance and hyperinsulinemia [7]. It has been proposed that pregnancy- induced insulin resistance reveals the onset of β-cell defects associated with insulin resistance and hyperglycemia, hyperlipidaemia [21]. which underlie GDM. GDM is diabetes restricted to pregnant women in whom the development of glucose intolerance was first diagnosed during pregnancy, distinguishing it from preexisting glucose intolerance and diabetes induced by pregnancy [21, 22]. This study was aimed at assessing placental peptides and maternal factors as potential predictors of the development of gestational diabetes among pregnant women.

The pre-gestational diabetic pregnant women had significantly higher plasma glucose, insulin and insulin resistance than the non diabetic pregnant women (Table 1).The plasma glucose and insulin resistance in the non diabetic pregnant women were significantly low probably due to the early stage of pregnancy. However, it has been shown that insulin resistance is associated with normal pregnancy and may not develop into GDM [23]. The mechanisms leading to insulin resistance in normal pregnancy are explained by several theories including; the estrogen and progesterone hormone counter effect on insulin action by their inhibitory effect on the skeletal muscle β-subunit of the insulin receptor and insulin receptor substrate-1[23] and thereby making available enough glucose for the growing foetus.[24]. These changes in insulin signaling reduces insulin-mediated glucose uptake in the muscle, the major tissues for whole-body glucose disposal [25]. However, insulin resistance is terminated soon after delivery and normal glucose metabolism returns within 1 year postpartum [26].

This phenomenon of insulin resistance in normal pregnancy was observed in this study. There was no significant difference, in plasma insulin, insulin resistance, β-HCG (P = 0.436)) and the lipid profile between pregnant women diagnosed with GDM and pregnant women with no diabetes at the early weeks of gestation (Table 2). This seems to suggest that, these peptides and the lipids may not play significant roles in the aetiology of GDM.

However, as the gestation progresses the effect of placenta peptides; Progestrone, estradiol, leptin and glycated haemoglobinon insulin predisposes the women to glucose intolerance and eventually to overt GDM [27, 28, 29]. Progestrone, and estradiol were significantly (p<0.0001) increased in the cases compared to the controls (Table 1). Progesterone, estradiol and leptin were also significantly higher in women with GDM than women with low risk of developing GDM (Table 3). This phenomenon may account for the role of these placenta peptides in the aetiology of GDM. Women with GDM have a combination of acquired and chronic insulin resistance and are more insulin resistant compared to the normal women during late pregnancy. The phosphorylation of insulin receptor tyrosine is implicated in the transmission of insulin signal to enable glucose uptake^30^. In women with GDM, there is a significant decrease in insulin receptor tyrosine phosphorylation in the skeletal muscle, and a decrease in insulin-stimulated glucose uptake [30]. Therefore, pregnancy can unmask slight defects that induce GDM. It has been proposed that these peptides interfere with insulin phoshorylation during pregnancy and impair glucose uptake [29].

The mean plasma leptin in the non diabetics pregnantwomen was nonsignificantly raised (Table 1), probably due to the smaller number of the women (12/150) who developed GDM had high leptin levels. This assertion is verified in Table 3, where leptin levels in GDM women is significantly higher than in women with low risk of GDM. Leptin concentration in amniotic fluid at 15–17 weeks of gestation is higher in women who subsequently developed GDM than in women with normal glucose tolerance during pregnancy [31]. Leptin enhances peripheral insulin sensitivity and improves pancreatic β-cell function [32].These normal functions of leptin are disrupted in leptin resistance state of pregnancy inducing insulin resistance and impaired glucose tolerance in pregnancy [33,34]. The placenta produces leptin during pregnancy resulting in further hyperleptinaemia. This is exacerbated by hyperleptinaemia due to fat accumulation, and subsequent insulin resistance, giving rise to GDM in women who are unable to compensate for the physiological dysregulation.

Anthropometric changes have been implicated in the aetiology of GDM. BMI was significantly (p **=** 0.022) higher in GDM women compared to pregnant women with low risk of GDM (Table 3). The risk of developing gestational diabetes increased with overweight (OR = 1.76, P = 0.370) (Table 5). Similar findings reported by Chu *et al*., shows that the risk of developing GDM is about 2 and 4 times higher among overweight and obese women respectively, as compared to normal-weight pregnant women [35].

Aging is a major confounding factor associated with the development of GDM. Even though the odd ratio of developing GDM due to ageing was low (OR = 1.02, P = 0.757) (Table 5), women within the age range of 30–39 had the highest probability of developing GDM (58%), whilst those at 20yrs and below had the lowest probability of 0.0%. (Fig 1). As women advance in age, there is a tendency to increased BMI, adiposity, multiparity and high blood pressure, and these are all confounding factors of GDM[36].

Apart from age, and BMI, other confounding factors that predisposes pregnant women to the development of GDM, include, family history of diabetes, ethnicity, adiposity and parity [37]. In a North American population, higher rates of GDM were reported among blacks, Hispanics, and Asians compared to non-Hispanic whites [38, 39]. A family history of diabetes increased the odds of developing GDM (OR = 2.18, P = 0.282). Pregnant women with a history of abortion, miscarriage and pre-mature birth as complications had higher risk of developing GDM (Table 5).The risk of developing GDM increased with increasing plasma insulin (OR 1.04 P = 0.256) and progesterone (OR 1.04 P = 0.05). Also the placental peptides were significantly higher in women who developed GDM than those who had low risk for the development of GDM (Table 3). Progestrone also had a strong sensitivity and specificity in diagnosing GDM, whilst β-HCG, estradiol and HPL were poor predictors of GDM.

Glycated haemoglobin, fasting blood glucose and fasting plasma insulin, are the key markers of diabetes. None of the placenta peptides and hormones had a significant specificity and selectivity as a marker of GDM in the first trimester. However, plasma glucose of diagnostic value of 5.6mmol/l had significant specificity and selectivity as marker of GDM in the second trimester (Table 4).

Body mass index was significantly and positively correlated with glycated haemoglobin (.702*). Fasting blood glucose had an inverse and strong significant correlation (-.801**) with insulin levels among GDMs (Table 6). Though, HbA1c, insulin and β-HCG are useful metabolites for diagnoses and monitoring of diabetes mellitus, yet had fair diagnostic tests values for GDM, since the AUC (95% CI) were >0.05. Glycosylated haemoglobin is not usually used clinically to diagnose GDM, it is however useful in diagnosing pre-existing diabetes in early pregnancy [40].

**Fig 2:**
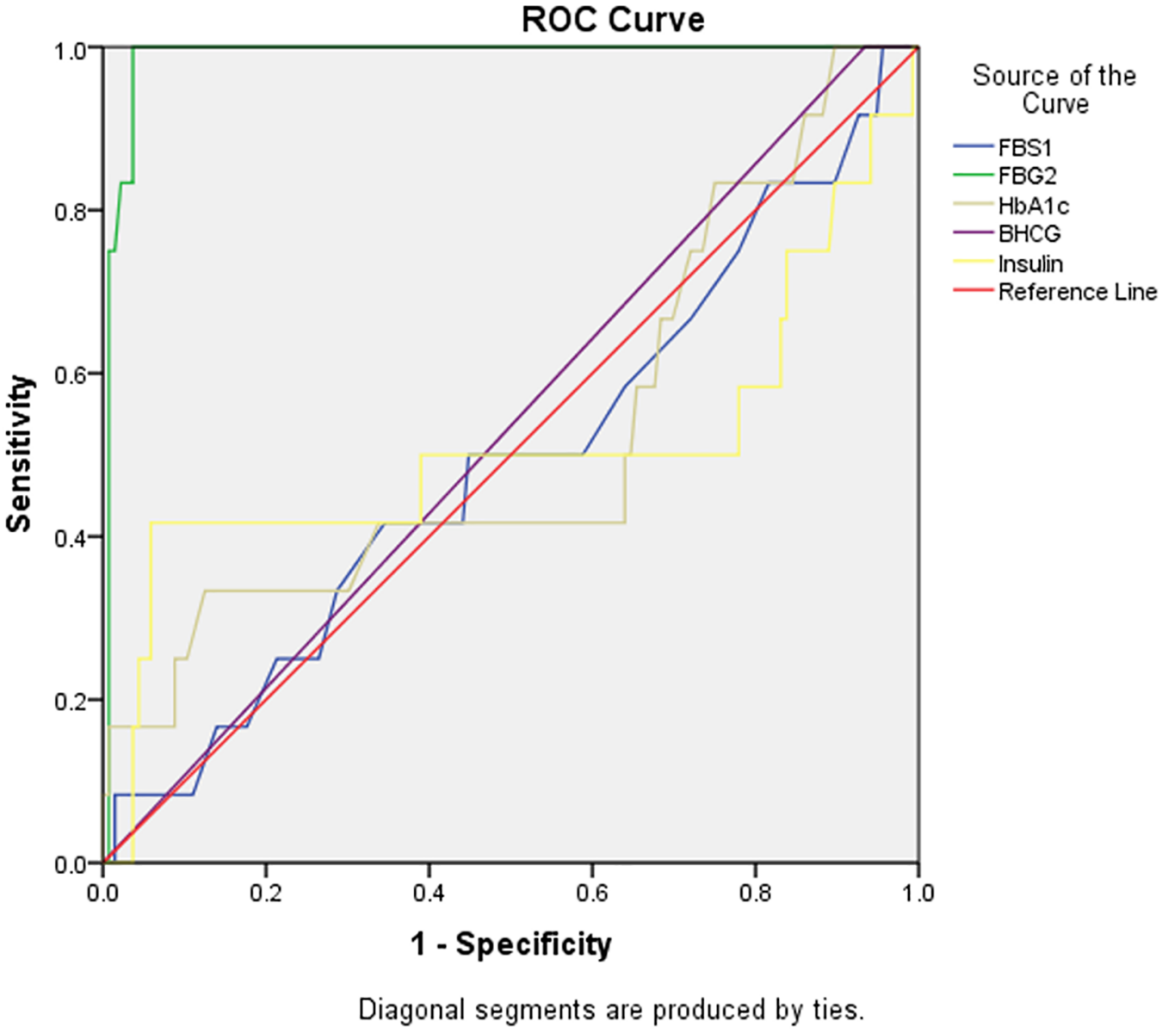
The receiver operator curve for fasting blood glucose, glycated haemoglobin, β-HCG and insulin levels. Receiver operator curve and area under the curve (AUC) 95% CI. The area under the ROC for FBG-1 (at first trimester) was 0.49. However, AUC for FBG-2 was F 0.99 (0.97–1.00), for week 24–28. HbA1c = 0.53 (0.33–0.72), Insulin = 0.51 (0.28), β-HCG = 0.53 (0.37–0.70).

The area under the ROC for FBG-1 (at first trimester) was 0.49 suggesting that fasting plasma glucose level in the first trimester is a poor diagnostic test for gestational diabetes mellitus. However, AUC for FBG-2 was 0.99 implying that FBG-2 is a good diagnostic test for diagnosing GDM between 24wks-28 wks, whereas, glycated haemoglobininsulin and β-HCG were poor diagnostic test markers for GDM as compared to FBS-2 (Fig 2).

## Conclusion

The crude prevalence of GDM in the metropolis was 8.0%.The plasma level of the placenta peptides, Leptin, Progesterone and Estradiol estimated were increased in the women who developed gestational diabetes mellitus. These peptides were fair in predicting GDM. However, progesterone level in the first trimester was outstanding in predicting the onset of GDM. Underweight, overweight, age and history of diabetes were strong predictors of GDM. HbA1c, insulin and β-HCG are fair tests for predicting GDM.

### Limitations

The effect of some confounding factors, ethnicity, adiposity on gestational diabetes were not independently determined.

## References

[1] Barbour LA, McCurdy CE, Hernandez TL, Kirwan JP, Catalano PM. and Friedman JE. Cellular mechanisms for insulin resistance in normal pregnancy and gestational diabetes. Diabetes care 2007; 30: 2S112–119 10.2337/dc07-s202 17596458

[2] King JC. Maternal obesity, metabolism, and pregnancy outcomes. Annu. Rev. Nutr. 2006; 26:271–291. doi: 10.1146/annurev.nutr.24.012003.132249 16704347 10.1146/annurev.nutr.24.012003.132249

[3] Buchanan T.A. and Xiang A.H. Gestational diabetes mellitus. The Journal of clinical investigation.2005: 115: 485–491. doi: 10.1172/JCI24531 15765129 10.1172/JCI24531 PMC1052018

[4] Wolf M, Sandler L, Hsu K, Vossen-Smirnakis K, Ecker JL, Thadhani R.First-Trimester C-Reactive Protein and Subsequent Gestational Diabetes Diabetes Care.2003;26:819–824, 12610043 10.2337/diacare.26.3.819

[5] Qiu C, Williams MA, Vadachkoria S, Frederick IO, Luthy DA. Increased maternal plasma leptin in early pregnancy and risk of gestational diabetes mellitus. Obstetrics & Gynecology 2004;103:519–25. 14990416 10.1097/01.AOG.0000113621.53602.7a

[6] Ramiya VK, Maraist M, Arfors KE, Schatz DA, Peck AB and Cornelius JG. Reversal of insulin-dependent diabetes using islets generated in vitro from pancreatic stem cells. Nature medicine 2000; 6: 278–282 doi: 10.1038/73128 10700229 10.1038/73128

[7] Ryan EA, O’Sullivan MJ, Skyler JS: Insulin action during pregnancy: studies with the euglycemic clamp technique. Diabetes.1985:34:380–389, 3882502 10.2337/diab.34.4.380

[8] Buchanan TA, Xiang A, Kjos SL and Watanabe R. What is gestational diabetes? Diabetes Care 2007; 30: S105–S111 doi: 10.2337/dc07-s201 17596457 10.2337/dc07-s201

[9] Kuhl C. Eatiology and pathogenesis of Gestational Diabetes Diabetes Care.1998; 21: B19–B21 9704223

[10] Hoffman L, Nolan C, Wilson JD, Oats J, Simmons D. Gestational diabetes mellitus—management guidelines: the Australasian Diabetes in Pregnancy Society. Med J Aust 1998;169:93–97 9700346 10.5694/j.1326-5377.1998.tb140192.x

[11] O'Sullivan J. The Boston Gestational Diabetes Studies In: Sutherland HW, Stowers JM, Pearson DWM, eds. Carbohydrate metabolism in pregnancy and the newborn. London: Springer-Verlag, 1989:287–94.

[12] Silverman B, Metzger BE, Cho NH, Loeb CA. Impaired glucose tolerance in adolescent offspring of diabetic mothers: relationship to fetal hyperinsulinism. Diabetes Care. 1995;18:611–617 8585997 10.2337/diacare.18.5.611

[13] Petitt D, Bennett PH, Knowler WC, Baird HR, Aleck KA. Gestational diabetes mellitus and impaired glucose tolerance during pregnancy: long-term effects on obesity and glucose intolerance in the offspring. Diabetes Care 1985;34:119–122 10.2337/diab.34.2.s119 3996763

[14] Rizzo TA, Metzger BE, Dooley SL, Cho NH. Early malnutrition and child neurobehavioural development: insights from the study of children of diabetic mothers. Child Dev. 1997;68:26–38 9084122

[15] American Diabetes Association. Standards of Medical Care in Diabetes—2013. Diabetes Care. 2013;36 (Suppl 1):S11–S66. 23264422 10.2337/dc13-S011 PMC3537269

[16] Crowther CA, Hiller JE, Moss JR McPhee AJ, William JS, and Robinson JS. Effect of Treatment of Gestational Diabetes Mellitus on Pregnancy Outcomes N Engl J Med. 2005; 352:2477–248 doi: 10.1056/NEJMoa042973 15951574 10.1056/NEJMoa042973

[17] Basevi V, Di Mario S, Morciano C, Nonino F, Magrini N. Comment on: American Diabetes Association. Standards of medical care in diabetes-2011. Diabetes Care. 2011;34:11–S61 10.2337/dc11-0174 PMC3114493 21525493

[18] Mamabolo RL, Alberts M, Levitt NS, Delemarre-van de Waal HA. and Steyn NP. Prevalence of gestational diabetes mellitus and the effect of weight on measures of insulin secretion and insulin resistance in third-trimester pregnant rural women residing in the Central Region of Limpopo Province, South Africa. Diabetic medicine: a journal of the British Diabetic Association.2007; 24: 233–239. 17263763 10.1111/j.1464-5491.2006.02073.x

[19] Lee AJ, Fanzca RJH, Franzcog PW, Franzcog SPW, Franzcog MP. Gestational Diabetes Mellitus: Clinical Predictors and Long-Term Risk ofDeveloping Type 2 Diabetes A retrospective cohort study using survival analysis Diabetes Care.2007;30:878–883, doi: 10.2337/dc06-1816 17392549 10.2337/dc06-1816

[20] Matthews DR, Hosker JP, Rudenski AS, Nalor BA, Treacher DF and Turner RC. Homeostasis model assessment: insulin resistance and β-cell function from fasting plasma glucose and insulin concentrations in man Diabetologia 1985; 28: 412–419 3899825 10.1007/BF00280883

[21] Sattar N, Greer I. Pregnancy complications and maternal cardiovascular risk: opportunities for intervention and screening? Br Med J 325:157–160, 2002 12130616 10.1136/bmj.325.7356.157 PMC1123678

[22] American Diabetes Association: Gestational diabetes mellitus. Diabetes Care. 2000;23: S77–S79,. 12017686

[23] Metzger BE, Buchanan TA, Coustan DR, De Leiva A, Dunger DB, Hadden DR et al Summary and Recommendations of the Fifth International Workshop-Conference on Gestational Diabetes Mellitus Diabetes Care. 2007; 30, S251–S260 doi: 10.2337/dc07-s225 17596481 10.2337/dc07-s225

[24] Lain KY and Catalano PM. Metabolic changes in pregnancy. Clinical obstetrics and gynecology. 2007; 50: 938–948 doi: 10.1097/GRF.0b013e31815a5494 17982337 10.1097/GRF.0b013e31815a5494

[25] Kelley DE, He J, Menshikova EV, and Ritov VB. Dysfunction of Mitochondriain Human Skeletal Muscle inType2Diabetes Diabetes. 2002 51: 2944–2950, 12351431 10.2337/diabetes.51.10.2944

[26] Coustan RD, Carpenter MW O'Sullivan PS, Carr RS. Gestational diabetes: Predictors of subsequent disordered glucose metabolism American Journal of Obstetrics and Gynecology.1993; 168: 1139–114 8475959 10.1016/0002-9378(93)90358-p

[27] Desoye G, and Hauguel-de Mouzon S, The Human Placenta in Gestational Diabetes Mellitus The insulin and cytokine network Diabetes Care.2007;30:S120–S126 doi: 10.2337/dc07-s203 17596459 10.2337/dc07-s203

[28] Handwerger S, Freemark M: The roles of placental growth hormone and placental lactogen in the regulation of human fetal growth and development.J Pediatr Endocrinol Metab 2000;13:343–356, 10776988 10.1515/jpem.2000.13.4.343

[29] Brelje TC, Scharp DW, Lacy PE, Ogren L, Talamantes F, Robertson M, Friesen HG, Sorenson RL: Effect of homologous placental lactogens, prolactins, and growth hormones on islet B-cell division and insulin secretion in rat, mouse, and human islets: implication for placental lactogen regulation of islet function during pregnancy. Endocrinology. 1993;132:879–887, doi: 10.1210/endo.132.2.8425500 8425500 10.1210/endo.132.2.8425500

[30] Friedman JE, Ishizuka T, Shao JL, Huston L Highman T and Catalano P Impaired glucose transport and insulin receptor tyrosine phosphorylation in skeletal muscle from obese women with gestational diabetes.Diabetes.1999; 48:1807–1814 10480612 10.2337/diabetes.48.9.1807

[31] D'Anna R, Baviera G, Corrado F, Ientile R, Granese D, Stella NC. Plasma homocysteine in early and late pregnancies complicated with preeclampsia and isolated intrauterine growth restriction. Acta obstetricia et gynecologica Scandinavica 2004;83:155–8. 14756732 10.1111/j.0001-6349.2004.00291.x

[32] Rabe K., Lehrke M., Parhofer K.G. and Broedl U.C. Adipokines and insulin resistance. Molecular medicin.2008; 14: 741–751 10.2119/2008-00058.Rabe PMC2582855 19009016

[33] Hauguel-de Mouzon S. and Guerre-Millo M. The placenta cytokine network and inflammatory signals. Placenta. 2006; 27: 794–798. doi: 10.1016/j.placenta.2005.08.009 16242770 10.1016/j.placenta.2005.08.009

[34] Hauguel-de Mouzon S, Lepercq J Catalano P. The known and unknown of leptin in pregnancy American Journal of Obstetrics and Gynecology. 2006; 194: 1537–1545 doi: 10.1016/j.ajog.2005.06.064 16731069 10.1016/j.ajog.2005.06.064

[35] Chu S.Y., Callaghan W.M., Kim S.Y., Schmid C.H., Lau J., England L.J. and Dietz P.M. Maternal obesity and risk of gestational diabetes mellitus. Diabetes Care. 2007; 30: 2070–2076 doi: 10.2337/dc06-2559a 17416786 10.2337/dc06-2559a

[36] Freinkel N, Metzger BE, Phelps RL, Dooley SL, Ogata ES, Radvany RM. Gestational Diabetes MellitusHeterogeneity of Maternal Age, Weight, Insulin Secretion, HLA Antigens, and Islet Cell Antibodies and the Impact of Maternal Metabolism on Pancreatic B-Cell and Somatic Development in the Offspring Diabetes, 1985; 34; 2: 1–7 10.2337/diab.34.2.s1 3888733

[37] Rodrigues S, Robinson EJ, Ghezzo H and Gray-Donald K Interaction of body weight and ethnicity on risk of gestational diabetes mellitus. Am J Clin Nutr 1999;70:1083–9. 10584054 10.1093/ajcn/70.6.1083

[38] Green JR, Pawson IG, Schumacher LB, Perry J, Kretchmer N. Glucose tolerance in pregnancy: ethnic variation and influence of body habitus. Am J Obstet Gynecol 1990;163:86–92. 2375375 10.1016/s0002-9378(11)90675-9

[39] Berkowitz GS, Lapinski RH, Wein R, Lee D. Race/ethnicity and other risk factors for gestational diabetes. Am J Epidemiol 1992;135:965–73. 1595695 10.1093/oxfordjournals.aje.a116408

[40] Sacks DB, Arnold M, Bakris GL, Bruns DE, Horvath AR, Kirkman MS, et al Guidelines and recommendations for laboratory analysis in the diagnosis and management of diabetes mellitus. Diabetes Care.2011; 34: e61–e99. doi: 10.2337/dc11-9998 21617108 10.2337/dc11-9998 PMC3114322

